# Preoperative thalamus volume is not associated with preoperative cognitive impairment (preCI) or postoperative cognitive dysfunction (POCD)

**DOI:** 10.1038/s41598-023-38673-x

**Published:** 2023-07-20

**Authors:** Marinus Fislage, Insa Feinkohl, Friedrich Borchers, Tobias Pischon, Claudia D. Spies, Georg Winterer, Norman Zacharias, BioCog Consortium, Alissa Wolf, Anika Müller, Daniel Hadzidiakos, Fatima Yürek, Gunnar Lachmann, Kwaku Ofosu, Maria Heinrich, Rudolf Mörgeli, Jürgen Gallinat, Simone Kühn, Arjen Slooter, Edwin van Dellen, Ilse Kant, Jeroen de Bresser, Jeroen Hendrikse, Simone van Montfort, David Menon, Emmanuel Stamatakis, Jacobus Preller, Laura Moreno-López, Stefan Winzeck, Daniela Melillo, Diana Boraschi, Giacomo Della Camera, Paola Italiani, Reinhard Schneider, Roland Krause, Karsten Heidtke, Peter Nürnberg, Anja Helmschrodt, Axel Böcher, Bettina Hafen, Franz Paul Armbruster, Ina Diehl, Jana Ruppert, Katarina Hartmann, Marion Kronabel, Marius Weyer, Thomas Bernd Dschietzig, Malte Pietzsch, Simon Weber, Bernd Ittermann, Ariane Fillmer

**Affiliations:** 1 Department of Anesthesiology and Operative Intensive Care Medicine (CCM, CVK), Charité–Universitätsmedizin Berlin, Corporate Member of Freie Universität Berlin and Humboldt-Universität zu Berlin, Berlin, Germany; 2 Faculty of Health/School of Medicine, Witten/Herdecke University, Witten, Germany; 3 Molecular Epidemiology Research Group, Max-Delbrueck-Center for Molecular Medicine in the Helmholtz Association (MDC), Berlin, Germany; 4 Biobank Technology Platform, Max-Delbrueck-Center for Molecular Medicine in the Helmholtz Association (MDC), Berlin, Germany; 5 Core Facility Biobank, Berlin Institute of Health at Charité - Universitätsmedizin Berlin, Berlin, Germany; 6 Pharmaimage Biomarker Solutions GmbH, Berlin, Germany; 7 Department of Anesthesiology and Operative Intensive Care Medicine (CCM, CVK), Charité–Universitätsmedizin Berlin, Corporate Member of Freie Universität Berlin and Humboldt-Universität zu Berlin, Berlin, Germany; 8 University Medical Center Hamburg, Hamburg, Germany; 9 University Medical Center Utrecht, Utrecht, The Netherlands; 10 University of Cambridge, Cambridge, UK; 11 National Research Council Napoli, Naples, Italy; 12 University of Luxembourg, Esch-sur-Alzette, Luxembourg; 13 ATLAS Biolabs GmBH, Berlin, Germany; 14 Immundiagnostik AG, Bensheim, Germany; 15 Cellogic GmbH, Berlin, Germany; 16 Physikalisch-Technische Bundesanstalt, Berlin, Germany

**Keywords:** Predictive markers, Cognitive ageing, Cognitive neuroscience, Neurological disorders

## Abstract

A growing body of literature suggests the important role of the thalamus in cognition and neurodegenerative diseases. This study aims to elucidate whether the preoperative thalamic volume is associated with preoperative cognitive impairment (preCI) and whether it is predictive for postoperative cognitive dysfunction at 3 months (POCD). We enrolled 301 patients aged 65 or older and without signs of dementia who were undergoing elective surgery. Magnetic resonance imaging was conducted prior to surgery. Freesurfer (version 5.3.) was used to automatically segment the thalamus volume. A neuropsychological test battery was administered before surgery and at a 3 month follow-up. It included the computerized tests Paired Associate Learning (PAL), Verbal Recognition Memory (VRM), Spatial Span Length (SSP), Simple Reaction Time (SRT), the pen-and-paper Trail-Making-Test (TMT) and the manual Grooved Pegboard Test (GPT). Using a reliable change index, preCI and POCD were defined as total Z-score > 1.96 (sum score over all tests) and/or Z-scores > 1.96 in ≥ 2 individual cognitive test parameters. For statistical analyses, multivariable logistic regression models were applied. Age, sex and intracranial volume were covariates in the models. Of 301 patients who received a presurgical neuropsychological testing and MRI, 34 (11.3%) had preCI. 89 patients (29.5%) were lost to follow-up. The remaining 212 patients received a follow-up cognitive test after 3 months, of whom 25 (8.3%) presented with POCD. Independently of age, sex and intracranial volume, neither preCI (OR per cm^3^ increment 0.81 [95% CI 0.60–1.07] p = 0.14) nor POCD (OR 1.02 per cm^3^ increment [95% CI 0.75–1.40] p = 0.87) were statistically significantly associated with patients’ preoperative thalamus volume. In this cohort we could not show an association of presurgical thalamus volume with preCI or POCD.

**Clinical Trial Number:** NCT02265263 (https://clinicaltrials.gov/ct2/show/results/NCT02265263)**.**

## Introduction

Depending on risk factors and study type, postoperative cognitive dysfunction (POCD) can be observed in 8.9% to 46.1% of surgical patients^1^. POCD is associated with increased mortality, prolonged necessity of social transfer payments and the premature termination of occupational practice^2^. POCD also causes substantial financial long-term care costs^3^. Hence, patients at risk should be identified and prevention strategies found. Little is known about the neural processes causing cognitive decline after surgery. Preoperative neuroimaging biomarkers may assist in risk stratification and allow insights into the neurobiological pathomechanisms leading to POCD. Previous research suggests that the thalamus might be a possible neuroimaging biomarker candidate for a different perioperative neurocognitive disorder: postoperative delirium (POD)^4^.

The thalamus is an important pharmacological target for most anesthetic agents which cause a reduction of thalamic blood flow and metabolism^5–7^. Anesthetics also affect thalamofrontal and resting neural connectivity in the anterior thalamic nuclei^8,9^. The structural integrity of the thalamus potentially mitigates the effect of stressors related to surgery such as anesthesia^10^. The brain reserve theory is the overarching theoretical concept underlying our research hypothesis^11–13^. In brief, it theorizes that a volumetric surplus of neurons helps individuals to cope with stressors, which may drive neurodegenerative processes. Older patients with a diminished thalamic cellular reserve may be particularly susceptible to perioperative cognitive disorders^14,15^.

While the crucial function of the thalamus as gatekeeper to consciousness, for instance during anesthesia, has been known for decades, its probable impact on cognition is receiving growing attention^14–17^. Although cognitive function has been predominantly linked to cortical regions^18^, recent cellular findings in mouse models have led to the assumption that the thalamus might play a role in coordinating rather than merely relaying cognitive processing. By recruiting inhibitory cortical neurons, the mediodorsal thalamus governs representation in the prefrontal cortex, which enables cognitive flexibility^19^. The pulvinar and the mediodorsal thalamus were shown to modulate the functional connectivity of cortical areas^20^. Moreover, cognitive domains such as declarative memory, executive functioning, attention, working memory and decision-making appear to rely on thalamic nuclei^16,21,22^.

Some epidemiological evidence suggests the thalamic function plays a role in age-related cognitive impairment. For instance, one study found that thalamic volume reduction was an early sign of amnestic mild cognitive impairment^23^. Strong thalamic volume reduction was also observed in Alzheimer’s disease^24^. In the perioperative setting, however, a study in middle-aged to older female patients with breast cancer found that a perioperative decline in thalamic grey matter did not coincide with an increased risk of POCD, which was operationalized as a decline in cognitive function from pre-surgery to a 6-day post-surgery assessment^25^.

A synopsis of prior research suggests that the thalamus might be a region of interest in the field of perioperative neurocognitive disorders. This secondary analysis was conducted as a longitudinal observational cohort study. We focused on preoperative brain health by measuring the preoperative thalamus volume in older patients scheduled for surgery by using structural magnetic resonance imaging. Our study objective was to investigate the possible association of presurgical thalamic volume with the presence of preoperative cognitive impairment (preCI) and its potential as a predictor for postoperative cognitive dysfunction at a 3-month follow-up (POCD). Furthermore, we aimed to clarify the role of the thalamus as a potential biomarker for perioperative neurocognitive disorders. Related findings may also help to understand the pathogenesis of cognitive impairment linked to surgical interventions. Our hypothesis suggests that a lower preoperative thalamus volume might be associated with preCI and it additionally predicts the onset of POCD.

## Materials and methods

This manuscript adheres to the applicable ‘Strengthening the Reporting of Observational Studies in Epidemiology’ (STROBE) guidelines^26^.

### Study setting and study population

**Figure 1:**
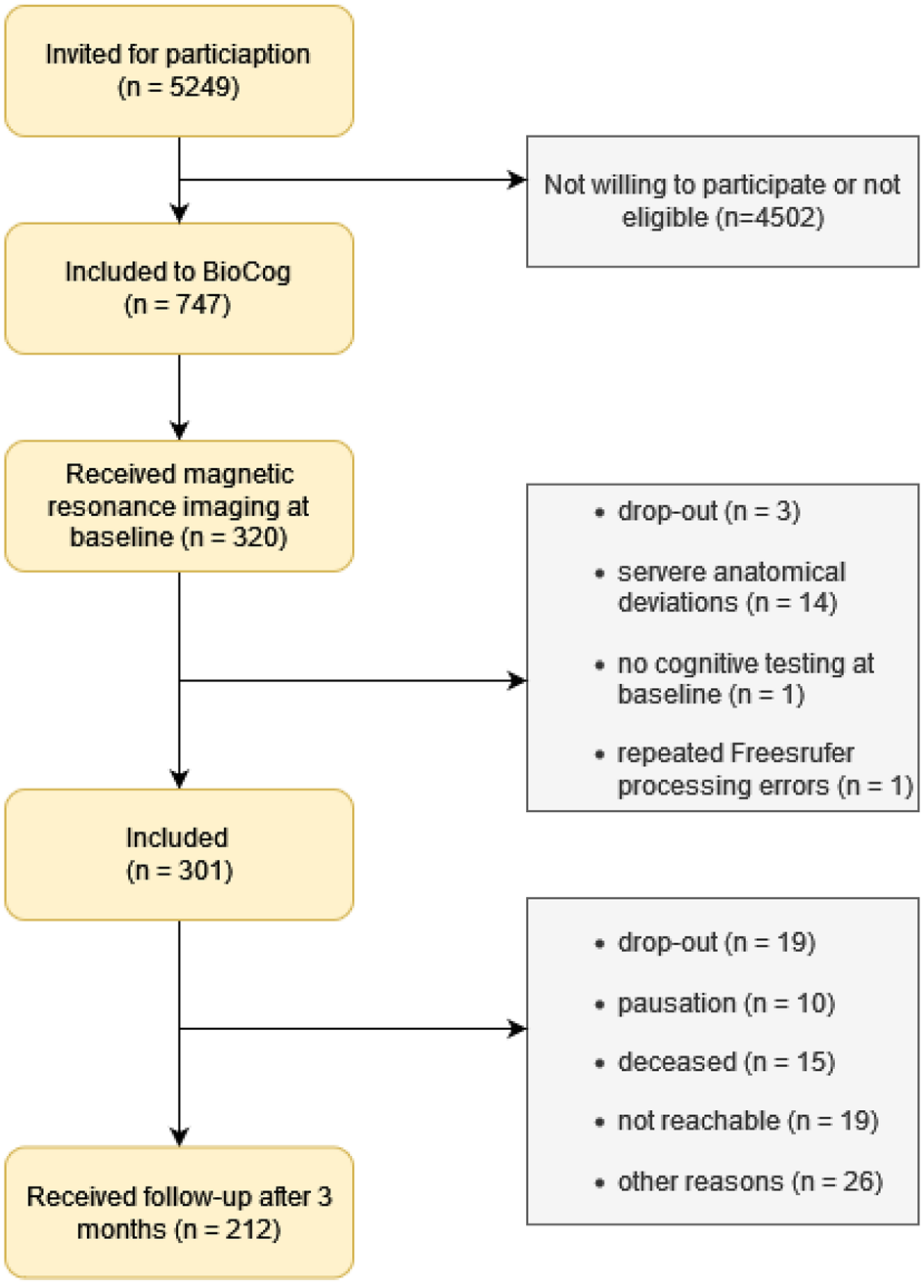
‘Strengthening the Reporting of Observational Studies in Epidemiology’ (STROBE) diagram. The flow chart shows reasons displays the inclusion process until the follow-up at 3 months. Reasons for exclusion are presented in gray boxes.

This exploratory secondary study is part of the ‘Biomarker Development for Postoperative Cognitive Impairment in the Elderly’ framework (BioCog; www.biocog.eu). The objectives and study design were previously published^27^. BioCog represents a multicenter prospective observational cohort study funded by the European Union. It was approved by local ethics committees (*Ethikkommission der Charité* No. EA2/092/14 in Berlin, Germany; and *Medisch Ethische Toetsingscommissie Utrecht* No. 14-469 in Utrecht, Netherlands) and was preregistered (NCT02265263). All methods were performed in accordance with all relevant guidelines and regulations that apply to research with human participants. The study was conducted in adherence with the Declaration of Helsinki. The patients’ written informed consent was obtained. Patients were enrolled from October 2014 to September 2019 at two study centers. To avoid test center effects, we exclusively included data from the MRI cohort of the Berlin study center, which was recruited at Charité—Universitätsmedizin Berlin, Germany. Our final analysis sample consisted of 301 patients (see Fig. 1).

Besides MRI eligibility, patients were deemed eligible, when they were aged > 65, did not show signs of dementia (Mini-Mental State Examination; MMSE > 23) and were assigned for major surgery (planned surgery time > 60 min). Any condition that might interfere with the interpretation of the individual neuropsychological test performance was a reason for exclusion, e.g., anacusis or hypacusis, blindness, psychiatric diseases, or psychotropic medication (https://clinicaltrials.gov/ct2/show/NCT02265263). For the patients’ characteristics see Table 1.

**Table 1:** Patient characteristics.

	All N = 301	No PreCI nor POCD N = 243	PreCI N = 34	POCD N = 25
Age [years]—mean (SD)	72.4 (4.9)	72.0 (4.8)	73.7 (4.4)	75.1 (6.0)
Female sex	131 (43.5%)	104 (42.8%)	17 (50%)	11 (44.0%)
Body mass index (BMI)—median (IQR)	26.6 (5.1)	26.5 (5.0)	26.8 (7.6)	27.1 (7.4)
PreCI	34 (11.3%)	–	–	1 (4.0%)
Postoperative delirium	44 (14.6%)	36 (14.8%)	7 (20.6%)	1 (4.0%)
POCD	25 (8.3%)	–	1 (2.9%)	–
Diabetes	72 (23.9%)	59 (24.3%)	7 (20.6%)	6 (24.0%)
Hypertension	203 (67.4%)	163 (67.1%)	22 (64.7%)	18 (72.0%)
History of stroke	13 (4.3%)	9 (3.7%)	3 (8.3%)	1 (4.0%)
Malignancy	115 (38.2%)	96 (39.5%)	12 (35.3%)	7 (28.0%)
Preoperative anaemia	81 (26.9%)	65 (26.7%)	9 (26.5%)	8 (23.0%)
Mini-mental state examination (MMSE)—median (IQR)	29 (2.0)	29 (2)	28 (2)	28 (1.1)
Benzodiazepine premedication	38 (12.6%)	29 (11.9%)	4 (11.8%)	5 (20.0%)
Duration of anesthesia [min]—mean (SD)	183.9 (116.3) N = 295	194.5 (120.7) N = 238	137.8 (88.8) N = 33	142.4 (74.6)
Type of anesthesia				
General	230 (76.4%)	178 (73.3%)	31 (91.2%)	22 (88.0%)
Regional	15 (5%)	13 (5.3%)	1 (2.9%)	1 (4.0%)
Combined	56 (18.6%)	52 (21.4%)	2 (5.9%)	2 (8.0%)
Type of surgery				
Musculoskeletal	85 (28.2%)	69 (28.4%)	9 (26.5%)	8 (32.0%)
Gastrointestinal	51 (16.9%)	45 (18.5%)	2 (5.9%)	4 (16.0%)
Cardiovascular or thoracic	17 (5.6%)	15 (6.2%)	1 (2.9%)	1 (4.0%)
Genitourinary	66 (21.9%)	54 (22.2%)	9 (26.5%)	3 (12.0%)
Otorhinolaryngology	23 (7.6%)	16 (6.6%)	6 (17.6%)	1 (4.0%)
Oral and maxillofacial	16 (5.3%)	10 (4.1%)	5 (14.7%)	1 (4.0%)
Ophthalmology	22 (7.3%)	18 (7.4%)	1 (2.9%)	3 (12.0%)
Neurosurgery	6 (2.0%)	3 (1.2%)	1 (2.9%)	2 (8.0%)
Other	15 (5.0%)	13 (5.3%)	0 (0%)	2 (8.0%)
ASA score				
ASA I	7 (2.3%)	6 (2.5%)	1 (2.9%)	0 (0%)
ASA II	204 (67.8%)	166 (68.3%)	23 (67.6%)	16 (64%)
ASA III	90 (29.9%)	71 (29.2%)	10 (29.4%)	9 (36%)
Length of stay [days]—median (IQR)	6 (6)	6 (6)	5.5 (5.25)	4 (6)
Inhouse mortality	5 (1.7%)	4 (1.6%)	1 (2.9%)	–
Follow-up at 3 months	212 (70.4%)	168 (69.1%)	20 (58.8%)	–
Thalamus volume [cm^3^]—mean (SD)	12.9 (1.6)	13.0 (2.5)	12.5 (1.3)	13.1 (1.6)
Intracranial volume [cm^3^]—mean (SD)	1339.0 (212.4)	1333.4 (207.9)	1321.6 (216.8)	1414.7 (239.4)

The table shows characteristics of all participants, the preoperative cognitive impairment (preCI) group and the postoperative cognitive dysfunction group (POCD) group. For categorical variables percentages are given instead of mean and standard deviation (SD) in parentheses. Percentages refer to the proportion of the corresponding group. The N of patients with available data was added in grey to items with cases of missing data. (ASA score ≙ American Society of Anesthesiologists’ Physical Status Classification; IQR ≙ Interquartile Range ≙ 25th to 75th percentile).

### Preoperative cognitive impairment (preCI) and postoperative cognitive dysfunction (POCD)

A neuropsychological test battery comprising four computerized (CANTAB, Cambridge Cognition Ltd., UK. Paired Associates Learning (PAL), Verbal Recognition Memory (VRM), Spatial Span Length (SSP) and Simple Reaction Time (SRT)) and two non-computerized cognitive tests (Trail-Making-Test (TMT) in a pen-and-paper format and the manual Grooved Pegboard Test (GPT)) was used for the cognitive assessment (Table 2). Study nurses and doctoral students were instructed according to a standard operating procedure that was developed by two neuropsychologists (Tables 3, 4).

**Table 2:** Neuropsychological tests.

Name of cognitive test	Task
Paired associates learning (PAL)—visuospatial memory	Different symbols appear in a randomized order in a distinct location on the screen. The symbols are then hidden, and participants are asked to show the location of a presented symbol. If they fail to remember the correct location on the screen, the task is repeated until a maximum of ten trials. In case of an accomplished sequence, there is a next level with an increased difficulty. As a result of the PAL, a memory score was calculated
Verbal recognition memory (VRM)—verbal memory and new learning	Participants must try to memorize a list of 12 words and repeat them freely afterwards. Then a second list was presented. It included the previous words and an additional number of distractors. Participants were asked to indicate, which items they remembered from the first list. After a delay of 20 min, the list appeared again. The number of recalled words was obtained and used for further analysis
Spatial span (SSP)—visual working memory	Color changing squares appeared in a random order on the screen. Participants were required to recall the order in which the squares have changed their color. The spatial length, which is the longest correct recognition sequence of in different order appearing squares, was measured
Grooved pegboard (GP)—visual-motor coordination	There are 25 pegs, which must be rotated so they fit into the differently shaped keyholes and could be correctly inserted. Participants were required to use only one hand. The time needed to accomplish the task with the dominant hand was used here
Simple reaction time (SRT)—reaction time	A square appeared on the screen. The time intervals were varying. Participants were instructed to select the response button as fast as possible after a square was shown. Here, we focused on the number of correct trials
Trail-making test (TMT)—cognitive flexibility, working memory and attention	In part A participants ought to draw a line between 25 numbers in a numerical order. Part B required the participants to draw a line alternating between numbers in a chronological and letters in an alphabetical order (‘START’ 1, A, 2, B…12, L, 13, ‘END’). The time needed to complete the task was measured. Lifting the pencil from the paper whilst being tested was not supposed to happen in neither of both tests. If the task had not been finished after 180 (part A) or 300 (part B) seconds, it was terminated and excluded from our analysis

For more information, please see Lammers et al.^28^.

**Table 3:** Neuropsychological test results (baseline).

	No PreCI nor POCD N = 243	PreCI N = 34	POCD N = 25
Paired associates learning (PAL)—memory score calculated for the first trial	14.0 (4.2) N = 239	9.4 (4.5)	14.0 (3.5)
Verbal recognition memory (VRM)—number of correctly remembered words in ‘Free recall’	6.3 (1.9)	3.9 (1.8) N = 32	6.7 (1.8) N = 24
Verbal recognition memory—number of correct and incorrect responses in ‘delayed recognition’	21.8 (1.9) N = 208	19.1 (2.4) N = 27	22.4 (1.8) N = 18
Spatial span (SSP)—spatial length (longest correct recognition sequence of squares appearing in different order	4.9 (0.9) N = 241	4.0 (0.9)	4.6 (1.0)
Grooved pegboard (GP) [s]	93.1 (24.7) N = 230	133.3 (43.7) N = 32	93.5 (19.9) N = 22
Simple reaction time (SRT) [s]—mean of correct trials (log-transformed and reversed)	309.0 (89.6) N = 242	412.0 (151.8)	322.6 (88.4)
Trail-making test B (TMT) [s]	112.3 (39.1) N = 222	174.5 (73.0) N = 24	125.6 (64.0) N = 22

The table displays mean and standard deviation in parentheses for preoperative cognitive test results. In case of missing data, the N of patients with available data was added to items with cases of missing data.

**Table 4:** Neuropsychological test results (post).

	No PreCI nor POCD N = 168	PreCI N = 20	POCD N = 25
Paired associates learning (PAL)—memory score calculated for the first trial	15.5 (3.9) N = 164	10.6 (4.8) N = 18	10.7 (4.7) N = 24
Verbal recognition memory (VRM)—number of correctly remembered words in ‘Free recall’	6.4 (1.7) N = 167	4.5 (1.6) N = 19	5.2 (1.4)
Verbal recognition memory—number of correct and incorrect responses in ‘Delayed Recogniton’	21.8 (2.0) N = 164	19.3 (2.6) N = 17	20.0 (2.6) N = 24
Spatial span (SSP)—spatial length (longest correct recognition sequence of squares appearing in different order	5.0 (0.8) N = 166	4.6 (1.1) N = 18	4.6 (0.8)
Grooved pegboard (GP) [s]	88.0 (16.7) N = 164	116.1 (25.0) N = 18	112.2 (37.5)
Simple reaction time (SRT) [s] mean of correct trials (log-transformed and reversed)	309.4 (77.0) N = 167	347.4 (94.9) N = 19	418.4 (184.1)
Trail-making test B (TMT) [s]	102.7 (35.0) N = 162	118.7 (30.3) N = 11	123.3 (48.5) N = 22

The table displays mean and standard deviation in parentheses for postoperative cognitive test results at follow-up. In case of missing data, the N of patients with available data was added to items with cases of missing data.

POCD was defined as a dichotomous variable based on an algorithm adjusting the difference in neuropsychological test scores between pre-surgery and a 3-month postsurgical assessment for natural variability and learning effects based on cognitive testing performed in a non-surgical control group a. For calculations the following seven cognitive test parameters were used^28^:

1. Paired Associates Learning—memory score calculated for the first trial.
2. Verbal Recognition Memory—number of correctly remembered words in ‘Free recall’.
3. Verbal recognition memory—number of correct and incorrect responses in ‘Delayed Recognition’.
4. Spatial span—spatial length (longest correct recognition sequence of squares appearing in different order).
5. Grooved pegboard—time (s) needed for the insertion of certain amount of pegs into differently-shaped holes on a board using the dominant hand (log-transformed and reversed).
6. Simple reaction time (s)—the mean of correct trials (log-transformed and reversed).
7. Trail-making test B (s)—(log-transformed and reversed).

To define relevant cognitive change and for dichotomization the Reliable Change Index model as published by Rasmussen et al.^29^ was then applied. POCD was defined as total Z-score > 1.96 (sum score over all tests) and/or Z-scores > 1.96 in ≥ 2 individual cognitive test parameters. We calculated PreCI using the same approach. To do so, we used patients’ preoperative neuropsychological data. The BioCog non-surgical control group included n = 114 participants. The stability of the neuropsychological tests was previously ascertained and published^30^. Furthermore, we have assessed the differences between surgical patients and the non-surgical control group (see Supplements). There were no statistically significant differences in terms of age, sex, body mass index and MMSE. However, the prevalence of comorbidities was lower among controls.

### Imaging

A 3 Tesla magnetic resonance imaging scanner (Siemens Trio Magnetom) was used to obtain structural brain images. The imaging sessions were hosted by the Berlin Center for Advanced Neuroimaging (BCAN; Berlin, Germany). We ran a T1-weighted 3D magnetization-prepared rapid gradient echo (MP RAGE) sequence (TR = 2500 ms, echo time = 4.77 ms, flip angle = 7°, 192 sagittal slices, field of view = 256 × 256 mm^2^, voxel size = 1 × 1 × 1 mm^3^). A 32-channel head coil was used. After image acquisition, a trained neuroradiologist examined the MRI data to identify intracranial pathologies.

Freesurfer (version 5.3.) on Linux CentOS6 (× 86) was used to automatically segment subcortical volumes. The processing of T1 weighted images included motion correction, averaging, removal of non-brain tissue compartments and Talairach transformation^31,32^. Subcortical structures were automatically identified and labeled^33^. Segmentation in Freesurfer proved to be as robust as manual delineation^34^. In particular, the thalamus volume can be reliably determined with this method^34^. Segmentation results were nevertheless manually reviewed. However, automatically assigned labels were not corrected by the reviewer since manual correction was decided to have little to no benefit^35^. Manual correction also negatively affects the reproducibility of the volumetric results. Severe anatomical deviations were excluded.

Volumetric measures were given in cubic millimeters. Freesurfer values for the left and the right thalamus hemisphere were combined to obtain a single variable for the entire thalamus. The Freesurfer variable ‘EstimatedTotalIntraCranialVol’ served as a measure for intracranial volume. (https://surfer.nmr.mgh.harvard.edu/fswiki/MorphometryStats).

### Statistical analysis

The scaling of volumetric data was adjusted from cubic millimeters to cubic centimeters. Statistical significance was defined as p < 0.05. Multicollinearity was assessed with the variance inflating factor (VIF) per variable. Multicollinearity was assumed at VIF > 2.5. Baseline missing-data were considered to be missing at random. The sample size for this specific analysis was not predetermined. However, general sample size calculations were undertaken for neuroimaging biomarkers in BioCog (see Supplements).

For the analysis of preCI and POCD, we ran a logistic regression model for each outcome. The accuracy of logistic regression models was determined using the area under the curve (AUC) of a receiver operating characteristic (ROC) curve. An AUC above 0.7 indicated a sufficient predictive value.

In this study, we intended to elucidate the role of the thalamus volume. Hence, thalamus volume was set as the predictor variable. We report unadjusted and adjusted odds ratio (OR). Adjustment covariates were integrated into the logistic regression based on their dependence structure prior to the statistical analysis. Since preCI was determined analogically to the definition of POCD, we used the same covariates for the preCI regression. For POCD, higher age was presented as a risk factor^36^. Similarly, thalamic volumes decrease with aging. Hence, the regressions measuring POCD included the variables age alongside thalamus volume. Brain atrophy might act as a potential confounder upon thalamus volume and POCD onset. Instead of brain atrophy, intracranial volume was described to be the variable appropriate for reflecting the cognitive ability in aging people^37^. Therefore, we also adjusted for intracranial volume.

To account for potential effects from the surgical procedure, we undertook a post-hoc sensitivity analysis, where we further included the surgery severity (minor, moderate, major and major+), type and duration of surgery. Moreover, we performed another sensitivity analysis using composite z-scores of cognitive data. The z-scores were calculated for baseline and follow-up data based on 928 surgical patients enrolled in the BioCog study. We also analyzed the change in z-scores from pre- to postoperative. Three linear regression models contained the thalamus as variable of interest and the respective z-scores as dependent variable. We again adjusted for age, sex and intracranial volume.

We used Graphpad Prism (Version 9.3.1 GraphPad Software, Inc.) for the statistical analysis and for creating graphs.

## Results

In total, 301 patients underwent neuropsychological testing and MRI before surgery. The mean age was 72.4 years (SD 4.9) and 131 (43.5%) were female (Table 1).

Of the 301 patients, 34 (11.3%) had preCI. Patients with preCI had a mean age of 73.7 years (SD 4.4) and 17 (50%) were female. Of the 34 patients who had preCI, 7 (20.6%) developed POD and 20 (58.8%) participated in the follow-up cognitive testing. We observed an OR of 0.79 ([95% CI 0.61–1.004] p = 0.06) per cm^3^ increment in thalamic volume when associated with preCI without further adjustment. After adjusting for age, sex and intracranial volume, the logistic regression model did not reveal any statistically significant association of thalamus volume with preCI (OR per cm^3^ increment 0.81 [95% CI 0.60–1.07] p = 0.14) (see Supplements). The area under the ROC curve was 0.60 (p = 0.04) (see Supplements). According to the calculated VIFs, multicollinearity was not present (see Supplements). The composite z-score of baseline cognitive tests was statistically significantly associated with the thalamus [Beta 0.15 (95% CI 0.06–0.23) p < 0.001].

Of the 212 patients that received the postoperative testing at the 3 month follow-up, 25 (11.8%) presented with POCD. Of the 89 patients (29.5%) that were loss-to-follow-up, 19 (6.3%) dropped out of the study, 15 (5.0%) died before the follow-up, 19 (16.3%) were not reachable, and 26 (8.6%) were still alive, but were not tested for different reasons. 10 patients (3.3%) paused their participation. Although they did not want to participate in the 3 month testing, they consented to attending the subsequent follow-up testing. Of the 89 patients that did not receive a cognitive assessment at the 3 months follow-up, 24 (27.0%) developed POD, 5 (5.6%) died during their postoperative stay in the hospital.

**Figure 2:**
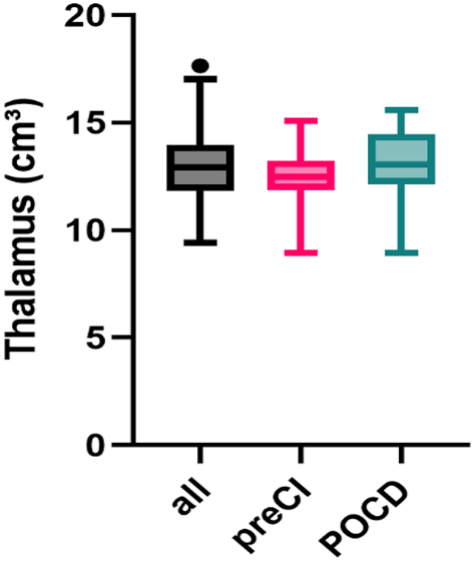
Boxplots of thalamus volume across groups. Thalamus volume in cm^3^ is displayed on the y-axis, while the different groups are placed on the x-axis: the entire cohort analysed in this study (n = 301 (all) in black, patients with preoperative cognitive impairment (preCI) in pink and patients with postoperative cognitive dysfunction (POCD) in green. Coloring was selected according to colorblind safe standards.

Of those 25 patients with POCD, one (4%) had preCI prior to, and one (4%) developed POD after surgery. The mean age of patients with POCD was 75.1 years (SD 6.0) with 11 (44.0%) female patients. In a simple logistic regression, thalamic volume was not statistically significantly associated with POCD (OR per cm^3^ increment 1.04 [95% CI 0.79–1.35] p = 0.79). After adjusting for covariates, the thalamus presented with an OR of POCD per cm^3^ increment of 1.02 (95% CI 0.75–1.40; p = 0.87) (see Supplements). The area under the ROC curve was 0.67 with a p-value = 0.005 (see Supplements). Multicollinearity was not observed (see Supplements). For the visualization of group differences see Fig. 2. After adjusting for the extent of surgery, we still did not observe an effect of thalamic volume on POCD (OR per cm^3^ increment 0.89 [95% CI 0.62–1.29] p = 0.54; n = 210). Using continuous postoperative z-scores and the change scores left the results unchanged (see Supplements).

## Discussion

In this exploratory secondary analysis of an observational cohort study in older patients we did not find an association of thalamus volume with preCI nor POCD. Thus, we presume that the preoperative thalamus volume is not a suitable biomarker. In accordance with the growing body of literature indicating a pivotal role of the thalamus in cognition, we could observe an effect of thalamic volume on preoperative cognition measured as continuous composite z-score.

While a smaller, possibly atrophic thalamus puts patients at risk for or can be observed in instances of mild cognitive impairment, Alzheimer’s disease and postoperative delirium^4,23,24^, this might not be the case in preCI and POCD. Perhaps the brain reserve theory cannot be directly applied to those instances of perioperative cognition. Although we have found an association of thalamic volume with preoperative cognition, this finding does not directly translate into a clinically relevant association with preCI as defined in this study.

A different study group has shown a thalamic volume reduction after surgery^25^. However, this was not statistically significantly associated with POCD. Notwithstanding these findings, a longitudinal analysis of the BioCog data may lead to different results. Separately, the POCD definition of this study differs profoundly from the BioCog definition since in this study POCD was determined at the seventh day after surgery^25^.

POCD as an outcome in research presents a variety of methodological shortcomings. For instance, definitions of POCD are fairly heterogenous^38^, which complicates comparing our findings in this outcome in particular with previous research. The “Recommendations for the Nomenclature of Cognitive Change Associated with Anaesthesia and Surgery” from 2018 suggests the term ‘delayed neurocognitive recovery’ for cognitive decline present 30 days after surgery^39^. After this period, experts recommend using the term ‘mild/major neurocognitive disorder postoperative’ for up to 12 months after surgery. The POCD definition conventionally used until this recommendation conflicts with the category of ‘mild/major neurocognitive disorder postoperative’, since the POCD follow-up was terminated at 3 months after surgical interventions. The newly proposed term still requires an additional assessment of the ‘activities of daily living’. Hence, we were not able to simply reassess our POCD variable, which was defined at the design stage of the BioCog study in 2016. This complicates the comparability with future studies.

### Limitations

This study faces further limitations. The patients more susceptible to developing POCD due to experiencing severe postoperative complications or suffering from a significant disease were, for these same reasons, more likely not to attend the 3 month follow-up. This may lead to an inherent selection bias within the follow-up cohort. Therefore, we probably underestimate the true number of patients with POCD. Patients who experienced major complications after surgery such as postoperative delirium are underrepresented in our POCD evaluation. For instance, only one patient with POCD (4%) had also experienced POD in the early days after surgery. This does not appear plausible considering the POD incidence of 44 (14.6%) for the whole analysis sample. The loss to follow-up was also higher than expected. The sample size was not predefined. We cannot rule out that this may not have caused insufficient statistical power. We recommend a detailed analysis with further independent surgical cohorts.

The relatively low POCD incidence might also be a direct consequence of a relatively strict cut-off of 1.96 in the reliable change index model defining relevant cognitive change. Applying an RCI method could also have caused other issues in the study^40,41^. The method used in this paper to determine POCD was published in 2001^29^. It was the generally preferred method when BioCog was designed, but just like the changes in terminology, the understanding of the very nature of POCD has evolved. For instance, some researchers recommend understanding perioperative neurocognitive disorders as a continuous change in cognitive performance rather than a dichotomous entity^42^. Another limitation regards the non-surgical control group, which was used to correct for learning effects the composition of the control group. Although the control group resembles the surgical study group in important demographic factors (e.g., age, sex, body mass index and MMSE score), both groups differ significantly in terms of comorbidities.

The anesthesiologic management was not standardized. However, to avoid the effect of deep anesthesia and high burst suppression rates all study participants were monitored with an intraoperative electroencephalogram (Masimo Sedline) according to the routine clinical treatment standard. We were not able to account for potential confounders that arose from the anesthesiologic handling.

Volumetric analyses can be affected by a variety of external and transient factors such as diurnal fluctuations, medication and hydration status^43^. We were not able to account for these factors.

## Conclusion

A relationship between thalamus size and preCI or POCD was not observed in our sample. These findings suggest that the thalamus volume does not predict cognitive function as defined in this study in older patients, neither before nor after surgery. Our findings indicate that the thalamus may not be involved in the etiology of preCI and POCD. Otherwise, its impact might not be adequately depicted by volumetric analyses. Future studies may require bigger sample sizes. Alternative analysis algorithms to handle raw cognitive data may also be needed.

## Supplementary Information

Supplementary Information.

## Data Availability

The raw data are under restricted access and can be requested via the EBRAINS repository (https://search.kg.ebrains.eu/instances/Dataset/09f0d6e2-b492-41b0-bba4-37ad9e54de27).
