# Supplementary material for: Preoperative thalamus volume is not associated with preoperative cognitive impairment (preCI) or postoperative cognitive dysfunction (POCD)
Source: Sci Rep. 2023 Jul 20;13:11732. doi: 10.1038/s41598-023-38673-x (PMC10359451; doi:10.1038/s41598-023-38673-x)
Supplement: Supplementary file 1 — Supplementary Information. [file 41598_2023_38673_MOESM1_ESM.docx]

|  | **All**  **N = 301** | **Controls**  **N=114** | **p-value** |
| --- | --- | --- | --- |
| Age [years] – mean (SD) | 72.4 (4.9) | 72.3 (6.1) | 0.83 |
| Female Sex | 131 (43.5%) | 56 (49.1%) | 0.31 |
| Body Mass Index (BMI) – median (IQR) | 26.6 (5.1) | 26.1 (5.0) | 0.23 |
| Mini-Mental State Examination (MMSE) – median (IQR) | 29 (2.0) | 29 (2.0) | 0.42 |
| Diabetes | 72 (23.9%) | 17 (15.0%) | 0.01 |
| History of Stroke | 13 (4.3%) | 8 (7%) | 0.57 |
| Malignancy | 115 (38.2%) | 6 (5.3%) | < 0.001 |
| **ASA score**   - ASA I - ASA II - ASA III | 7 (2.3%)  204 (67.8%)  90 (29.9%) | N = 113  22 (19.3%)  61 (53.5%)  30 (26.3%) | < 0.001 |
| Charlson Comorbidity Index –  Median (IQR) | 1 (2) | 0 (1) | < 0.001 |

SUPPLEMENTAL TBALE 1 **Characteristics of patients vs. controls**

*Note:* The table shows characteristics of all participants versus the non-surgical control group. For categorial variables percentages are given instead of mean and standard deviation (SD) or median and interquartile range (IQR ≙ 25th to 75th percentile) in parentheses. Percentages refer to the proportion of the corresponding group. The N of patients with available data was added in grey to items with cases of missing data. A two-tailed t-test was used for parametric data, whereas we applied a median test for non-parametric data. We used the chi²-test to asses significant differences between categorial variables. (ASA score ≙ American Society of Anesthesiologists’ Physical Status Classification)

SUPPLEMENTAL TABLE 2 **Multivariable logistic regression – Preoperative Cognitive Impairment**

|  | *Odds ratio* | *95% Confidence Interval* | *p-value* |
| --- | --- | --- | --- |
| **Thalamus [cm³]** | **0.81** | **0.60 - 1.07** | **0.14** |
| Intracranial Volume [cm³] | 1.00 | 0.998 - 1.00 | 0.87 |
| Sex | 0.96 | 0.40 - 2.32 | 0.93 |
| Age [year] | 1.04 | 0.97 - 1.12 | 0.26 |

Note: Table showing the results of a multivariable logistic regression with odds ratio (OR) for preoperative cognitive impairment (preCI). Effect sizes are given as odds ratio (OR) per increment in units given in parentheses.

SUPPLEMENTAL TABLE 3 **Multivariable logistic regression – Postoperative Cognitive Dysfunction**

|  | *Odds ratio* | *95% Confidence Interval* | *p-value* |
| --- | --- | --- | --- |
| **Thalamus [cm³]** | **1.02** | **0.75 - 1.40** | **0.87** |
| Intracranial Volume [cm³] | 1.00 | 0.999 - 1.01 | 0.13 |
| Sex | 0.62 | 0.20 - 1.82 | 0.38 |
| Age [year] | 1.11 | 1.02 - 1.21 | 0.02 |

Note: Table showing the results of a multivariable logistic regression with odds ratio (OR) for postoperative cognitive dysfunction (POCD). Effect sizes are given as odds ratio (OR) per increment in units given in parentheses.

SUPPLEMENTAL TABLE 4 **Multicollinearity assessment with variance inflating factor (VIF)**

| *Preoperative Cognitive Impairment* | *Variance Inflating Factor (VIF)* | *R² with other variables* |
| --- | --- | --- |
| Thalamus [cm³] | 1.398 | 0.2849 |
| Intracranial Volume [cm³] | 1.612 | 0.3796 |
| Sex | 1.549 | 0.3542 |
| Age [year] | 1.087 | 0.0804 |

| *Postoperative Cognitive Dysfunction* | *Variance Inflating Factor (VIF)* | *R² with other variables* |
| --- | --- | --- |
| Thalamus [cm³] | 1.335 | 0.2510 |
| Intracranial Volume [cm³] | 1.583 | 0.3683 |
| Sex | 1.529 | 0.3460 |
| Age [year] | 1.100 | 0.0912 |

SUPPLEMENTAL FIGURE 1 **Receiver Operating Characteristics (ROC) curves**

**
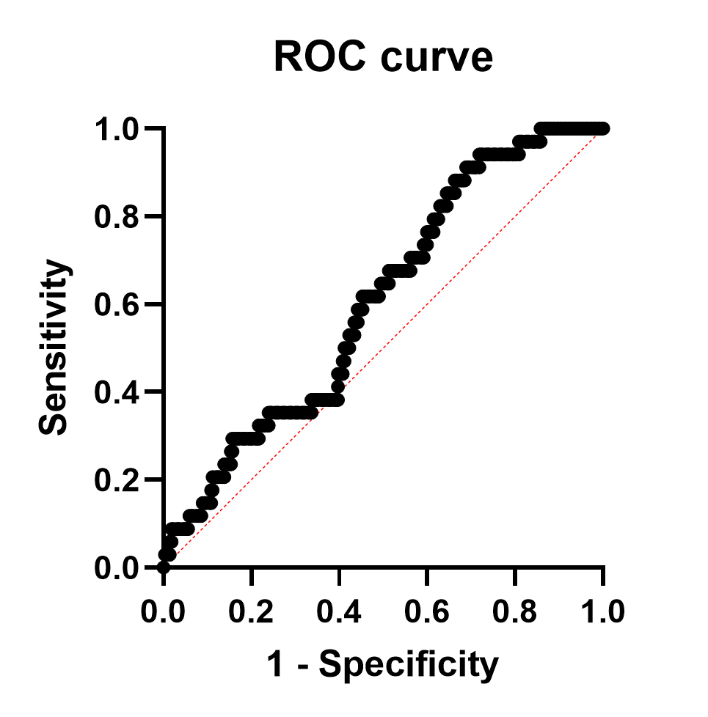
**

Note: ROC curve for the logistic regression model with the thalamus as predictor variable and preoperative cognitive impairment (preCI) as dependent variable. The area under the ROC curve was 0.60 (p=0.04).


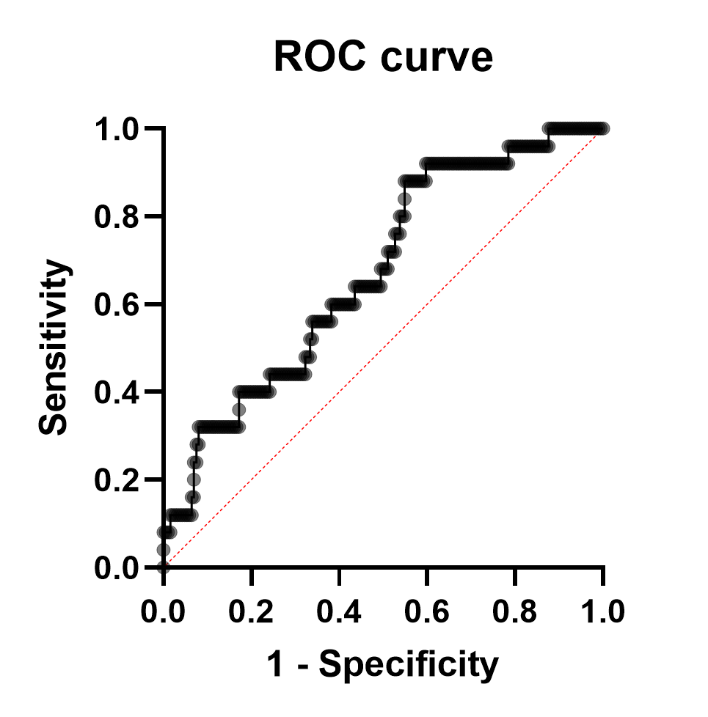


Note: ROC curve for the logistic regression model with the thalamus as predictor variable and postoperative cognitive dysfunction (POCD) as dependent variable. The area under the ROC curve was 0.67 with a p-value = 0.005

SUPPLEMENTAL TABLE 5 **Multivariable linear regressions – continuous z-scores**

| *Thalamus Volume* | *Beta* | *95% Confidence Interval* | *T* | *p-value* |
| --- | --- | --- | --- | --- |
| ***Preoperative*** | **0.15** | **0.06 -0.23** | **3.48** | **< 0.001** |
| *Postoperative* | 0.04 | -0.11 – 0.18 | 0.47 | 0.64 |
| *Change Score* | 0.93 | -0.12 – 0.30 | 0.87 | 0.38 |

Note: Table showing the regression coefficients of the thalamus volume in three different multivariable linear regression models (preoperative, postoperative, change score). The regression models were adjusted for age, sex and intracranial volume. The change score was derived from the difference in baseline to postoperative z-scores.

SUPPLEMENTAL INFORMATION **Sample Size calculation**

Assuming an incidence for a perioperative neurocognitive disorder of 20-30% and a 10% drop-out rate, an effect size of Hedges G 0.5 to yield 80% power (α=5%, two-sided) roughly requires a sample size of 200 patients for any imaging biomarker.
